# 

**Published:** 1890-04

**Authors:** 


### ASSOCIATION NOTICES.

### American Medical Association.

The forty-first annual session will be held in Nashville, Tenn., on Tuesday, Wednesday, Thursday and Friday, May 20, 21, 22 and 23, commencing on Tuesday at 11 A.M.

"The delegates shall receive their appointment from permanent organized State Medical Societies, and such County and District Medical Societies *as are recognized by reprentation in their respective State Societies*, and from the Medical Department of the Army and Navy, and the Marine-Hospital Service of the United States.

"Each State, County and District Medical Society entitled to representation shall have the privilege of sending to the Association one delegate for every ten of its regular resident members, and one for every additional fraction of more than half that number; *provided*, however, that the number of delegates for any particular State, territory, county, city or town shall not exceed the ratio of one in ten of the resident physicians who may have signed the Code of Ethics of the Association."

*Members by Application.*—Members by Application shall consist of such members of the State, County and District Medical Societies entitled to representation in this Association as shall make application to the Treasurer and accompany said application with a certificate of good standing, signed by the President and Secretary of the society of which they are members, and the amount of the annual membership fee, five dollars. They shall have their names upon the roll, and have all the rights and privileges accorded to *permanent members*, and shall retain their membership upon the same terms.

The following resolution was adopted at the session of 1888:

"That in future each delegate or permanent member shall, when he registers, also record the name of the Section, if any, that he will attend, and in which he will cast his vote for Section officers."

Secretaries of Medical Societies as above designated are earnestly requested to forward, *at once*, lists of their delegates.

Also, that the permanent Secretary may be enabled to erase from the roll the names of those who have forfeited their membership, the Secretaries *are, by special resolution*, requested to send him annually a corrected list of the membership of their respective societies.

AMENDMENTS TO THE BY-LAWS.

Offered by Dr. A. L. Gihon, United States Navy.

That the first day of the meeting of this Association shall be on the first Wednesday of May or June respectively, instead of Tuesday.

By Dr. X. C. Scott, Ohio:

That the Committee on State Medicine be abolished, inasmuch as the Section on State Medicine occupies the entire ground.

ADDRESSES.

On General Medicine, by Dr. N. S. Davis, Chicago, Ill.

On General Surgery, by Dr. Samuel Logan, New Orleans, La.

On State Medicine, by Alfred L. Carroll, New York, N. Y.

Committee of Arrangements: Dr. William T. Briggs, Nashville, Tenn.

WILLIAM B. ATKINSON, Perm. Sec'y.

---

THE Supreme Court of Georgia has decided that the proprietor of a patent medicine is liable for damages for the injury done to any person who takes the medicine according to directions.

### Programme of the Dental Section.

The forty-first annual session of the American Medical Association will be held in Nashville, Tenn., on Tuesday, Wednesday, Thursday and Friday, May 20, 21, 22, and 23, commencing Tuesday at 11 A. M.

The following are the subjects of papers to be read before the dental section:

Relation of Tropho-Neurosis to Diseases of the Mouth and Jaws with Special Reference to Syphilitic Necrosis, by ...... DR. G. FRANK LYDSTON.

How the Vascular Supply is Connected with the Teeth, by ...... DR. A. O. HUNT.

Vascular Tumors of the Mouth, and Treatment by Injection, by ...... DR. JOHN S. MARSHALL.

Electro-Therapeutics, by...... DR. JOHN L. GISH.

The Value of Illustration in the Lecture Room, by ...... DR. MCINTOSH.

Adenoid Growths and their Effect on the Mouth, by ...... DR. E. E. BRIGGS.

Cure for Cleft Palate by a Double-Flap Operation and Closure with the Buried Tendon Suture, by...... DR. H. O. MARCEY.

Diseases of the Gums and their Treatment, by...... DR. J. TAFT.

Irregularities of the Teeth Caused by Neurotic Conditions, by ...... DR. E. S. TALBOT.

Hereditary Dental Anomalies, by...... DR. WM. S. SHERMAN.

### International Medical Congress of 1890.—Special Notice.

All those who propose to attend the session of the International Medical Congress in Berlin next August are requested to forward to the undersigned their names and the number of tickets they will require. Arrangements are now in progress to obtain special rates on the lines to Europe. For the Commitee,

WM. B. ATKINSON, M.D.,
Permanent Sec'y Am. Med. Ass'n.

### Report of the Committee on Foreign Transportation.

To the Editor:—The Committee on Foreign Transportation have finally arranged as follows:

The Netherlands–American Steam Navigation Co. being the only line that would allow any reduction sufficient to be worth mentioning and on times of sailing suitable for our delegates agree to give us transportation to Boulogne, France, or Rotterdam, Amsterdam, Holland, from New York and return for $90 in first-class rooms inside or $105 outside, this *only* to *our own members* and *their families.*

Sailing from New York in May, June or July, as follows:

May 1st, 8th, 15th, 22nd, 29th, June 5th, 12th, 19th, 26th, July 3rd, 10th, 17th. Berths for May steamers must be engaged before April 15th; June, by May 1st; and July by May 15th. After these dates the company may dispose of all berths on said steamers not engaged. Of course those who secure births first get the best places. All the steamers are alike in first-class accommodations.

For those who desire it, free passage will be granted from Rotterdam, Amsterdam via Flushing-Queensboro' to London on the outward trip. The boats running between Flushing Queensboro' carry the mail of Northern Europe to and from Great Britain. This route is the most direct and favorite one from London to Berlin, and the steamers are all elegantly furnished. There is a day and night service, the boats running in the night all have state rooms.

For reserving berths on the steamer from New York a deposit of $10 each person is required. The balance of the passage money must be paid at least two weeks prior to sailing.

These steamers cross in eleven to twelve days, some do it in less time.

Railroad fare from Rotterdam to Berlin, 1st class $13.60; 2nd class, $10.40; Amsterdam to Berlin, 1st, $13.70; 2nd, $10.50. Boulogne to Paris, 1st, $6.00; 2nd, $4.50.

The table and attendance on the steamers to be of the best.

Efforts are being made to obtain a reduction in the railroad fares as above. This will be announced in the *Journal* as soon as obtained.

(Signed) WM. H. PANCOAST, Chairman of Com.
W. B. ATKINSON, Sec'y.

Those who have already notified the secretary of their intention to go to Berlin will in due time receive the proper certificate which will entitle them to these rates. Those who have not sent their names are requested to do so at an early date.

Yours truly, WM. B. ATKINSON.

Philadelphia, Feb. 20th, 1890.

### Tenth International Medical Congress—Preliminary Programme of the Dental Section.

Monday, the 4th of August, 1890, after the close of the first general session; constitution of the dental session by the election of officers.

From Tuesday 5th until Saturday 9th inclusive practical demonstrations will be given each day in the forenoon, between 9 and 12 o'clock, in the Dental Institute of the Royal University, Dorotheenstrasse 40.

These demonstrations will consist in extraction and narcosis, in filling and in artificial work. Gentlemen desiring to demonstrate either in extraction and narcosis or in artificial work are requested to apply to Professor Busch, (Alexander-Ufer 6). Those who wish to demonstrate in filling are requested to apply to Professor Miller (Vossstrasse 32). For these demonstrations there are in the dental institute 15 chairs with good light, which number in case of urgent necessity might be increased to 19. The afternoons, between 2 and 5 o'clock, will be devoted to theoretical lectures and discussions, in the hall of the Ressource zur Unterhaltung, Oranienburgerstr 18.

The following five themes have been selected, the discussion of which will be opened by members appointed for the purpose.

1. Narcosis with bromide of ethyl in dental operations.
2. The cause, course and treatment of pyorrhœa alveolaris.
3. The participation of micro-organisms in decay of the teeth.

4. Crown and bridgework.
5. The Bonwill method of articulation.

The members are also at liberty to present communications upon subjects with which they have particularly occupied themselves. Such communications along with a short resume of their contents should be announced to the Chairman of the Committee, Professor Busch, (Alexander-Ufer 6) On those days on which general sessions are held (between 11—2 o'clock) the practical demonstrations will be closed earlier and the theoretical lectures begun later.

### EDITORIAL.

### THE DENTAL REGISTER,

A MONTHLY MAGAZINE DEVOTED TO THE INTERESTS OF THE

**DENTAL PROFESSION.**

*Terms Reduced to $2.00 Per Year. Single Copies, 25 Cents.*

**EDITED BY PROF. J. TAFT, D.D.S.**

—AND—

**Published by SAM'L A. CROCKER & CO., Ohio Dental and Surgical Depot.**

The volume commences in January and closes in December.

The REGISTER being issued monthly is always fresh, therefore Indispensable to the practitioner who desires to keep posted up with the new inventions and improvements which are daily developed. Neither expense nor effort will be spared to give value and variety to its contents. Articles will be illustrated with well executed engravings whenever they will add to the interest or assist to explanation.

Its extensive circulation and frequency of issue make it one of the BEST DENTAL ADVERTISING MEDIUMS in the United States.

All subscriptions must begin with January or July.

Local or special notices, five lines or less, will be inserted for $3 each insertion; over five lines, 50 cts. per line.

All advertising bills payable in advance, or at furthest, after the issue of the first number containing the advertisement. All transient advertisements to be paid for in advance.

☞CONTRIBUTIONS SOLICITED.

Exclusively Editorial Communications should be addressed to the Editor.

The latest number of the REGISTER may be ordered with or without other goods at any time, and all letters should be addressed to

**SAM'L A. CROCKER & CO., Ohio Dental and Surgical Depot, Cincinnati, O.**

